# Diagnostic Value of the Neutrophil/Lymphocyte Ratio, Platelet/Lymphocyte Ratio, and Thrombocytosis in the Preoperative Investigation of Ovarian Masses

**DOI:** 10.1055/s-0040-1712991

**Published:** 2020-06-19

**Authors:** Adriana Yoshida, Luís Otavio Sarian, Marcos Marangoni, Isis Caroline Firmano, Sophie Françoise Derchain

**Affiliations:** 1 Department of Obstetrics and Gynecology, Faculty of Medical Sciences, Universidade Estadual de Campinas, Campinas, São Paulo, Brazil

**Keywords:** adnexal masses, diagnosis, ovarian cancer, serum biomarkers, thrombocytosis, massas anexiais, diagnóstico, câncer de ovário, biomarcadores séricos, trombocitose

## Abstract

**Objective** To evaluate the diagnostic accuracy of cancer antigen 125 (CA125) and complete blood count (CBC) parameters, such as the neutrophil to lymphocyte ratio (NLR), the platelet to lymphocyte ratio (PLR), and thrombocytosis in patients with ovarian masses.

**Methods** The present is a retrospective study conducted at a single tertiary hospital from January 2010 to November 2016. We included consecutive women referred due to suspicious adnexal masses. The CBC and CA125 were measured in the serum of 528 women with ovarian masses before surgery or biopsy. We evaluated the diagnostic performance of the NLR, PLR, platelets (PLTs), CA125, and the associations between them. We tested the clinical utility of the CBC parameters and CA125 in the discrimination of ovarian masses through decision curve analysis (DCA).

**Results** The best balance between sensitivity and specificity was obtained by the associations of CA125 or PLTs ≥ 350/nL, with 70.14% and 71.66%, CA125 or PLTs ≥ 400/nL, with 67.30% and 81.79%, CA125 or PLR, with 76.3% and 64.87%, and CA125 or NLR, with 71.09% and 73.89% respectively. In the DCA, no isolated CBC parameter presented a higher clinical utility than CA125 alone.

**Conclusion** We showed that no CBC parameter was superior to CA125 in the prediction of the malignancy of ovarian tumors in the preoperative scenario.

## Resumo

**Objetivo** Avaliar a acurácia diagnóstica do antígeno de câncer 125 (cancer antigen 125, CA125, em inglês) e dos parâmetros do hemograma como as razões neutrófilo/linfócito (RNL), plaqueta/linfócito (RPL), e trombocitose em pacientes com massas ovarianas.

**Métodos** Este é um estudo retrospectivo realizado em um hospital terciário no período de janeiro de 2010 a novembro de 2016. Foram incluídas de forma consecutiva mulheres encaminhadas por massas anexiais suspeitas. Foram dosados hemogramas e CA125 no soro de 528 mulheres com massas ovarianas antes da cirurgia ou biópsia. Foram avaliados os desempenhos diagnósticos da RNL, da RPL, das plaquetas (PLQs) e do CA125, considerando-os isoladamente e associados entre si. Testamos a utilidade clínica dos parâmetros do hemograma e do CA125 na discriminação das massas ovarianas por análise de curva de decisão (ACD).

**Resultados** Os melhores equilíbrios entre sensibilidade e especificidade foram obtidos por meio das associações do CA125 ou PLQs ≥ 350/nL, com 70,14% e 71,66%, CA125 ou PLQs ≥ 400/nL, com 67,30% e 81,79%, CA125 ou RPL, com 76,3% e 64,87%, e CA125 ou RNL, com 71,09% e 73,89%, respectivamente.

**Conclusão** Na ACD, nenhum parâmetro do hemograma isolado se mostrou superior ao CA125 na predição de malignidade de tumores ovarianos no pré-operatório.

## Introduction

Ovarian cancer (OC) is the second most lethal gynecological cancer worldwide, although it is relatively rare.^1^ On the other hand, adnexal masses are frequently found on transvaginal ultrasonography or other imaging exams, and the diagnosis of these patients is challenging. The patients benefit from a more accurate preoperative diagnosis, since it may help in the medical decision-making regarding the adequate treatment or follow-up. The diagnostic accuracy of serum biomarkers has been evaluated, and although some approaches besides cancer antigen 125 (CA125) alone are commercially available, such as the risk of ovarian malignancy algorithm (ROMA, LabCorp, Burlington, NC, US) and OVA1 (Vermillion, Austin, TX, US), there is a gap of more simple and accessible tools to be widely used in a population with adnexal masses.

Since chronic inflammation plays a fundamental role in the pathogenesis of OC,^2^ several studies^3^ ^4^ ^5^ ^6^ ^7^ ^8^ have investigated systemic inflammatory response (SIR) markers obtained from a simple complete blood count (CBC), such as the neutrophil to lymphocyte ratio (NLR), the platelet to lymphocyte ratio (PLR), and the platelet (PLT) count. They have been evaluated as relevant prognostic factors in OC,^3^ ^4^ but a few studies^5^ ^6^ ^7^ ^8^ have focused their usefulness in the prediction of malignancy in the preoperative setting.

At the time of diagnosis, an elevated PLT count, namely thrombocytosis (PLT count ≥ 400/nL^9^ or ≥ 350/nL^7^), is found in women with ovarian cancer, and is associated to a decreased overall survival (OS) and poor prognosis.^4^ In the tumor and host tissues, there is an increased production of thrombopoietic cytokines (mainly IL-6 [Interleukin 6]), which leads to paraneoplastic thrombocytosis, resulting in tumor growth and progression.^10^ Moreover, the PLTs do not allow contact of the tumor cells with the host's immune system, preventing natural killer cell-mediated elimination of tumor cells and supporting the extravasation of tumor cells.^11^

In a recent systematic review^12^ and meta-analysis it was found that a high NLR was associated with decreased OS and event-free survival (EFS) in patients with gynecologic cancers (OC, cervical and endometrial). Studies have indicated that neutrophils inhibit the immune system and contribute to the creation of an inflammatory microenvironment, promoting tumor growth, vascularization and metastasis.^12^

Two meta-analyses^3^ ^13^ assessed the prognostic value of NLR and PLR for long-term survival (OS and progression-free survival [PFS]) in OC patients. Overall, the pooled data provided evidence that both higher NLR and higher PLR predicted inferior survival outcomes. However, there is a lack of data about the role of CBC parameters in the preoperative diagnosis of women with adnexal masses, which could be useful especially in the case of equivocal imaging on ultrasonography.

The present study aimed to investigate if there is a role for these parameters (alone or combined with CA125) in the preoperative discrimination of ovarian tumors. Our hypothesis is that CBC parameters, especially the PLR and thrombocytosis, might be better predictors of malignancy than isolated CA125.

## Methods

The present is a retrospective study that evaluated women from January 2010 to November 2016 at the women's hospital (Centro de Atenção Integral à Saúde da Mulher, CAISM, in Portuguese) of Universidade Estadual de Campinas, a tertiary cancer center specialized in gynecological malignancies. The university's Ethics in Research Committee approved the study (under number 1092/2009). The eligibility criteria were women referred to the pelvic oncology clinic due to adnexal masses detected on ultrasound or another imaging exam, who had a CBC before they were submitted to surgery or percutaneous biopsy. The patients were consecutively included after signing a consent form, and they were submitted to the study protocol. First, they were submitted to a physical exam, and a pelvic ultrasound was scheduled. Blood samples were collected for serum tumor marker (CA125) and CBC measurement. Although samples for the biomarker and CBC dosage were not collected at the same moment, both were done before surgery or diagnostic biopsy. Diagnostic and/or surgical procedures were scheduled after the evaluation of the pelvic ultrasound, the CA125 dosage and the physical exam.

**Fig. 1:**
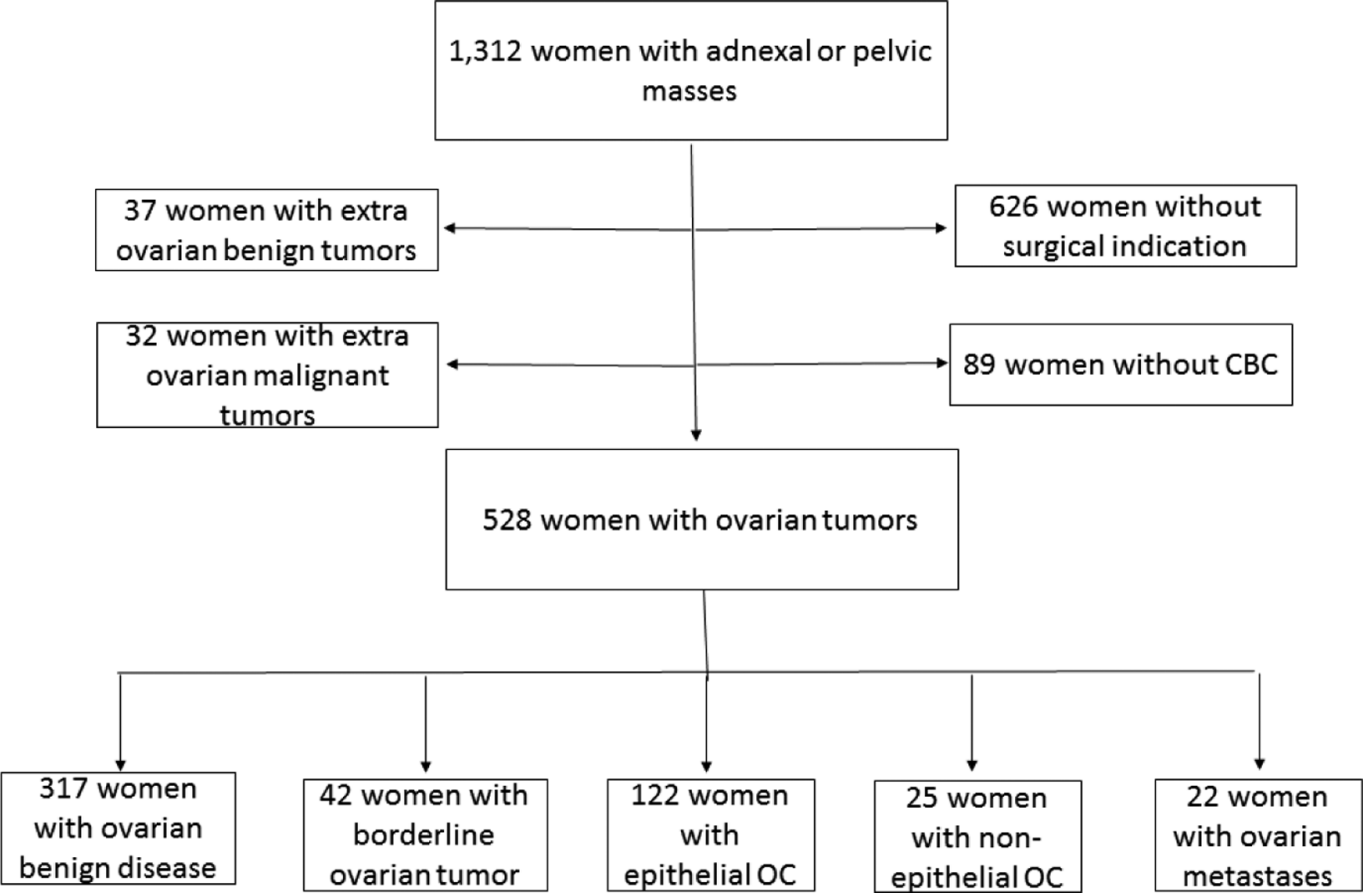
Flowchart of the women included in the study. Abbreviations: CBC, complete blood count; OC, ovarian cancer.

For this diagnostic accuracy study, the sample size was calculated based on the following parameters: expected specificity of 80%, with a delta of 15%, and study power of 90% (β error of 10%). In addition, we used 95% confidence intervals (95% CIs, *p* = 0.05), denoting an allowed α error of 5%. With these parameters, 85 patients with malignant ovarian tumors and another 85 patients without malignant ovarian tumors must be included. The calculations were based on the formulas provided in the appendix of Flahault et al,^14^ using the “power.diagnostic.test” function of the MKmisc library for the R Environment (R Foundation for Statistical Computing, Vienna Austria). In the present study, we enrolled 1,312 consecutive women, and we excluded those with no surgical indication (626 women), those without preoperative CBC (89 women), and those who had ovarian benign tumors (37 women), such as uterine leiomyoma, tubal cysts, sactosalpinx, and other ovarian malignant tumors (32 women), such as, endometrial cancer and carcinomas of gastrointestinal origin In total, 528 women with ovarian tumors were included (Fig. 1). Percutaneous biopsies of pelvic masses or abdominal implants were performed in cases of non-resectable ovarian tumors. Tissue specimens were analyzed by pathologists specialized in gynecologic pathology, according to the guidelines of the World Health Organization's (WHO) International Classification of Ovarian Tumors.^15^ For tumor staging, we followed the classification of the International Federation of Gynecologists and Obstetricians (FIGO).^16^ Bilateral tumors were found in 104 women; for the purpose of categorization, the tumor with worst prognosis was taken into account. Postmenopausal status was defined as > 1 year of amenorrhea or ≥ 50 years of age in the case of previous hysterectomy. Five women did not have a preoperative CA125 dosage, but were not excluded from the study. No patient underwent chemotherapy before surgery or biopsy of the tumor in our casuistic.

### CA125 Measurement

We collected blood samples from patients by puncture of a peripheral vein before surgery or percutaneous biopsy, and the samples were stored in serum separator tubes for the CA125 dosage. For the majority of the women, serum CA125 was determined by the CA125 II test, through the electrochemiluminescence technique in the Cobas e411 (Roche Diagnostics GmbH, Mannheim, Germany) automatic analyzer, according to the manufacturer's instructions. The other women had their serum CA125 concentrations measured by the Architect (Abbott Diagnostics, Abbott Park, IL, US) automated assay, a two-step chemiluminescent microparticle immunoassay (CMIA), according to the manufacturer instructions, or by solid-phase chemiluminiscence in an automated system, using the OM-MA (Siemens Medical Solutions Diagnostics, Tarrytown, NY, US) test, according to the manufacturer's instructions. All CA125 values were expressed in units per milliliter (U/mL). The levels of CA125 were considered elevated if ≥ 35 U/mL.

### Complete Blood Count

The CBC was obtained from patients before the surgery or the percutaneous biopsy were performed. If the patient had more than one preoperative CBC, we chose the nearest to the surgery/biopsy for analysis. The CBC was obtained through the automated hematology analyzer Sysmex XE-2100 (TOA Medical Electronics, Kobe, Japan) from 2010 until 2012, according to the manufacturer's instructions. After 2012, the analyzer used was the Sysmex XE 5000 (TOA Medical Electronics). According to our receiver operating characteristic (ROC) curve analyses, the optimal cutoff point for elevated PLR was considered if ≥ 150.9, and elevated NLR was considered if ≥ 2.9. Cutoff points for thrombocytosis (PLT ≥ 350/nL and PLT ≥ 400/nL) were obtained from large studies on the subject.^7^ ^9^

### Statistical Analysis

**Fig. 2:**
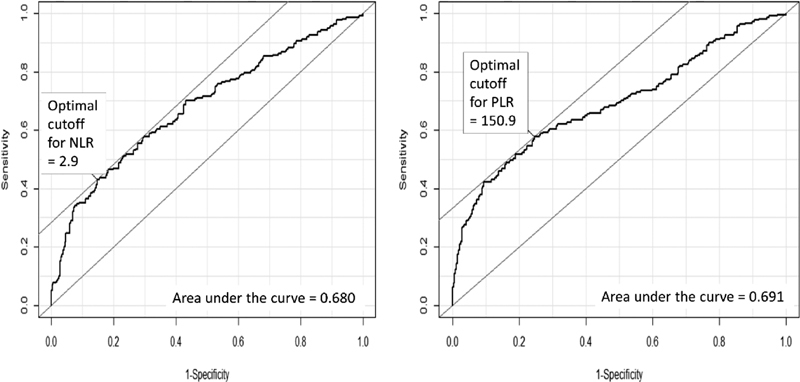
Analyses of the receiver operating characteristic (ROC) curve of the A) neutrophil to lymphocyte ratio (NLR) and B) platelet to lymphocyte ratio (PLR) for the diagnosis of malignant tumors in women with adnexal masses. The optimal cutoff points are shown in the figures, along with the performance indicators (sensitivity, specificity) and the areas under the curves.

The data were analyzed using the R Environment for Statistical Computing software (R Foundation for Statistical Computing).^17^ Statistical calculations were performed using 95% CIs, considering *p* values < 0.05 as significant. Ovarian tumors were classified into benign diseases (non-neoplastic and benign ovarian tumors), borderline ovarian tumor (BOT), epithelial ovarian cancer (EOC), non-epithelial ovarian cancer (non-EOC), and ovarian metastatic groups according to the histopathologic diagnosis, using the current criteria as proposed by Kurman el al.^15^ We determined the mean (with corresponding standard deviation [SD]) values for age, body mass index (BMI), serum CA125 levels, NLR, PLR and PLT levels, according to tumor groups. Data on BMI was not available for ten women. For further analysis, the BOT, EOC, non-EOC and ovarian metastatic groups were considered malignant. We then calculated the sensitivity, specificity, positive predictive value (PPV), negative predictive value (NPV), positive likelihood ratio (LR), negative LR, and accuracy for CA125, NLR, PLR, PLTs ≥ 350/nL, PLTs ≥ 400/nL, and the associations of 2 parameters (combinations were considered positive in different circumstances: when the CA125 and the CBC parameter were both positive, and when the CA125 or the CBC parameter were positive) to test the diagnostic performance for the prediction of malignancy. The cutoff values for the NLR and PLR (Fig. 2) were obtained from the ROC curve (NLR ≥ 2.9 and PLR ≥ 150.9). Finally, we performed decision curve analysis (DCA) to test which parameters or their associations presented the best clinical utility to differentiate benign from malignant masses. For this graphic analysis, we used software designed specifically for DCA.^18^ All DCA calculations were performed as described by Vickers and Elkin.^19^

## Results

Table 1 shows the clinical features of patients and their tumors, and the CA125 levels and CBC parameters of 528 women included in the study. Benign ovarian diseases comprised 27.4% of non-neoplastic ovarian lesions, and 72.6% of benign ovarian tumors. Among the malignant tumors, 19.9% were BOTs, 10.4% were ovarian metastases, 57.8% were EOCs, and 11.8% were non-EOCs. The total malignancy prevalence of our cohort was of 39.96%. Women with EOC had a mean age of 59.22 years (SD: 14.42 years), and those with ovarian metastases had a mean age of 53.45 years (SD: 11.08 years), while women with benign ovarian diseases, as well as those with BOTs and non-EOCs, had a mean age < 50 years. In total, 75% of the women with EOC were postmenopausal. Regarding the stage of the disease, it was initial for 83.3% of the women with BOTs, 47.5% of the women with EOCs, and 96% of those with non-EOCs.

**Table 1:** Clinical features of the women by type of tumor

	Benign‡ disease (*n* = 317)	Borderline ovarian tumor (*n* = 42)	Epithelial OC (*n* = 122)	Non-epithelial OC (*n* = 25)	Ovarian metastases (*n* = 22)
Age, in years†	46.79 (16.17)	48.37 (16.22)	59.22 (14.42)	43.58 (18.17)	53.45 (11.08)
Premenopausal, n (%)	177 (55.84)	20 (47.62)	30 (24.59)	14 (56.0)	11 (50)
Postmenopausal, n (%)	140 (44.16)	22 (52.38)	92 (75.41)	11 (44.0)	11 (50)
Body Mass Index, in Kg/m^2^ †	28.57 (5.79)	28.67 (5.49)	27.17 (5.76)	28.42 (6.51)	27.43 (4.93)
CA125, in U/ml†	67.39 (214.59)	167.55 (238.25)	1156.73 (3353.65)	156.13 (258.19)	532.68 (1469.04)
Platelets/nl†	269,712.9 (74,493.58)	285,023.8 (69,662.42)	339,606.6 (115,450.86)	337,520.0 (153,029.99)	360,727.3 (153,945.01)
Neutrophil to lymphocyte ratio†	2.20 (1.35)	2.87 (2.43)	3.81 (3.22)	2.63 (1.50)	9.43 (15.03)
Platelet to lymphocyte ratio†	131.08 (56.23)	170.46 (123.62)	217.17 (155.09)	183.69 (95.35)	311.09 (218.70)
**Disease stage**					
I-II, n (%)		35 (83.3)	58 (47.54)	24 (96)	
III-IV, n (%)		7 (16.7)	64 (52.46)	1 (4)	

Abbreviations: CA125, cancer antigen 125; OC, ovarian cancer.

Notes:

† Values expressed as mean (standard deviation).

‡ Benign disease involves benign ovarian tumors and ovarian non-neoplastic lesions.

Table 2 shows the preoperative diagnostic performance of CA125, PLT, NLR and PLR in a pool of women with ovarian masses. Overall, the isolated CBC parameters tested showed low sensitivity, but high specificity to differentiate benign from malignant ovarian tumors. The best balance between sensitivity and specificity was when we used CA125 or PLT ≥ 350/nL, with 70.14% and 71.66%, CA125 or PLT ≥ 400/nL with 67.30% and 81.79%, CA125 or PLR, with 76.3% and 64.87%, and CA125 or NLR, with 71.09% and 73.89% of sensitivity and specificity respectively. These associations showed a slight gain in sensitivity but a decrease in specificity when compared with isolated CA125. Moreover, thrombocytosis (PLT ≥ 400) alone, or associated to CA125, thrombocytosis (PLT ≥ 350) and PLR with a cutoff point of 150.9 associated to CA125 achieved specificities higher than 95%, in spite of low sensitivities. In addition, the PPV of the association of CA125 and PLT ≥ 350, PLT ≥ 400, and PLR were remarkably high: 91.18%, 95.45% and 85.85% respectively, whereas the PPV of CA125 alone was of 73.45%. Table 2 also lists the accuracy of the separate and combined tests for the differentiation of benign from borderline and OC (accuracy) and to discern benign from borderline and stage I OCs (accuracy 2. Please see the Table 2). The best accuracy was obtained with CA125 as a separate test, and for the restricted dataset (excluding diseases in stage II or higher), the best accuracy was obtained with the combination of CA125 ≥ 35 U/mL AND PLR ≥ 150.9.

**Table 2:** Preoperative diagnostic performances of CA125, PLT, PLR and NLR

	Sensitivity	Specificity	PPV	NPV	Positive LR	Negative LR	Accuracy	Accuracy 2*
CA125 ≥ 35 U/mL	61.90% (56.52 to 67.28)	84.98% (79.72 to 90.25)	73.45%	76.88%	4.12	0.45	75.72%	72.6%
PLT ≥ 350/nL	37.91% (32.57 to 43.25)	84.86% (78.65 to 91.07)	62.5%	67.25%	2.50	0.73	66.10%	69.1%
PLT ≥ 400/nL	25.59% (20.79 to 30.40)	96.21% (91.61 to 100.82)	81.82%	66.02%	6.76	0.77	67.99%	73.9%
PLR ≥ 150.9	57.82% (52.38 to 63.26)	75.08% (69.10 to 81.06)	60.70%	72.78%	2.32	0.56	68.18%	68.0%
NLR ≥ 2.9	42.65% (37.21 to 48.10)	85.17% (79.22 to 91.12)	65.69%	69.05%	2.88	0.67	68.18%	71.3%
CA125 ≥ 35 U/mL or PLT ≥ 350/nL	70.14% (65.08 to 75.20)	71.66% (65.92 to 77.39)	62.45%	78.12%	2.47	0.42	71.05%	66.4%
CA125 ≥ 35 U/mL or PLT ≥ 400/nL	67.30% (62.10 to 72.49)	81.79% (76.43 to 87.15)	71.36%	78.77%	3.69	0.40	75.95%	72.6%
CA125 ≥ 35 U/mL and PLT ≥ 350/nL	29.52% (24.49 to 34.55)	98.10% (94.86 to 101.34)	91.18%	67.68%	15.55	0.72	70.72%	75.2%
CA125 ≥ 35 U/mL and PLT ≥ 400/nL	20% (15.60 to 24.40)	99.37% (97.03 to 101.71)	95.45%	65.22%	31.7	0.80	67.74%	74.0%
CA125 ≥ 35 U/mL or PLR ≥ 150.9	76.30% (71.61 to 80.99)	64.87% (59.20 to 70.55)	59.19%	80.39%	2.17	0.36	69.45%	65.0%
CA125 ≥ 35 U/mL and PLR ≥ 150.9	43.33% (37.85 to 48.81)	95.22% (91.16 to 99.28)	85.85%	71.53%	9.07	0.59	74.43%	75.6%
CA125 ≥ 35 U/mL or NLR ≥ 2.9	71.09% (66.08 to 76.10)	73.89% (68.23 to 79.54)	64.66%	79.18%	2.72	0.39	72.77%	68.8%
CA125 ≥ 35 U/mL and NLR ≥ 2.9	33.33% (28.14 to 38.53)	96.20% (92.07 to 100)	85.36%	68.47%	8.8	0.69	71.10%	75%

Abbreviations: CA125, cancer antigen 125; LR, likelihood ratio; NLR, neutrophil to lymphocyte ratio; NPV, negative predictive value; PLR, platelet to lymphocyte ratio; PLT, platelet; PPV, positive predictive value.

Notes: The cutoff points for the PLR and NLR were derived from receiver operating characteristic (ROC) curve analysis (Fig. 2), because there are no standard points reported in current medical literature. The cutoff points for CA125 levels and PLT counts are those used as standard in the medical literature.

* Accuracy for the differentiation of benign masses from borderline or stage I ovarian cancer (stage II or higher not included in this analysis).

**Fig. 3:**
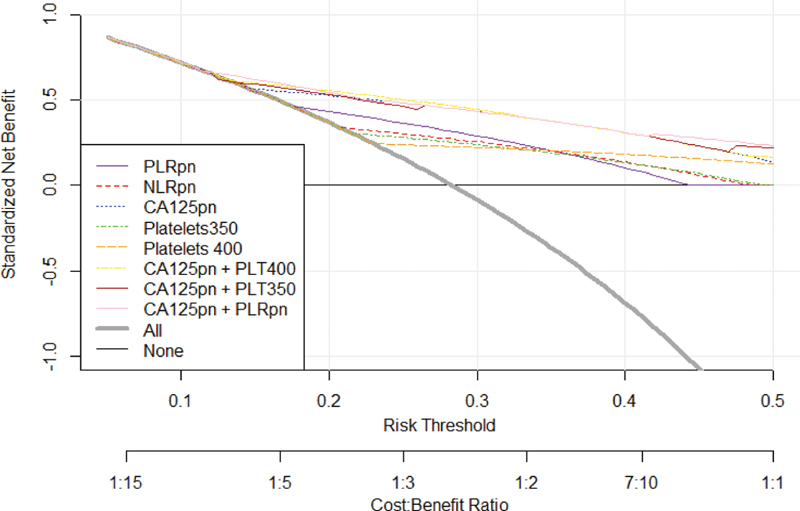
Decision curve showing the net benefit of complete blood count parameters isolated or in association with CA125 in women at risk of developing ovarian cancer, borderline ovarian tumor and ovarian metastases. PLRpn, platelet to lymphocyte ratio, positive if ≥ 150.9, negative if < 150.9; NLRpn, neutrophil to lymphocyte ratio, positive if ≥ 2.9, negative if < 2.9; CA125pn, CA125 dosage, positive if ≥ 35 U/mL, negative if < 35 U/mL; Platelets 350, platelets ≥ 350/nL; Platelets 400, platelets ≥ 400/nL.

Fig. 3 shows the DCA for the CBC parameters and CA125. This graphic analysis showed that no isolated CBC-derived parameter presented a higher clinical utility than CA125 alone. When we associated thrombocytosis (PLT ≥ 400/nL or PLT ≥ 350/nL) and PLR with a cutoff point of 150.9 to CA125, we observed an enhancement of this clinical value, which was higher than the isolated CA125 clinical value in the range of 15% to 25% risks thresholds.

## Discussion

In the present study, we evaluated and compared CBC parameters and the diagnostic accuracy of CA125 levels in a casuistic of 528 women with various histologic types of ovarian tumors. We showed that the NLR, the PLR or the thrombocytosis did not present a superior performance in the prediction of malignancy compared with CA125 dosage in the preoperative setting.

Chronic inflammation has been associated with the carcinogenesis of different types of tumors, including ovarian cancer (OC). This process occurs through different mechanisms such as inhibition of apoptosis, promotion of angiogenesis, stimulation of DNA damage, and up-regulation of cytokines and inflammatory mediators. In addition, SIR mediators impair the immune system, which leads to an increase in the concentrations of leukocytes, neutrophils, platelets, C-reactive protein (CRP) and fibrinogen, and a decrease in the levels of albumin and lymphocytes.^20^ ^21^

Differently from Watrowski et al,^7^ in the present study, CA125 alone presented lower sensitivity and specificity in the differentiation of malignant and benign ovarian tumors. It is of note that, in their study, CA125 was available in 314/756 (41.5%) of the included individuals. However, when they associated CA125 with thrombocytosis, the specificity (0.94 for PLT ≥ 350 and 0.96 for PLT ≥ 400) and the PPV (0.91 for both cutoff points of PLT) were as high as in the present study. We obtained for the CA125 and PLT ≥ 350 a specificity of 98.10%, with a PPV of 91.18%, and, for the CA125 and PLT ≥ 400, a specificity of 99.37%, with a PPV of 95.45%, although the sensitivity in the present study was < 30% for both associations. In addition, in the study by Watrowski et al,^7^ thrombocytosis alone could improve the preoperative diagnosis of adnexal masses, but, in the present study, DCA showed that isolated thrombocytosis, PLR or NLR presented lower net benefit than CA125 alone or CA125 combined to the evaluated CBC parameters.

The NLR alone, with a cutoff of 2.9, presented a low sensitivity (42.65%), but a better specificity (85.17%), in the prediction of malignancy in the present study. Moreover, DCA showed a low net benefit for NLR across the percentages of risk threshold. The cutoff points vary in the published studies, making it difficult to compare our results. Eo et al^8^ showed that the NLR (cutoff of 2.64), the PLR (cutoff 1of 91.71), elevated platelets, the CA125 and the lymphocyte-monocyte ratio (LMR) were able to predict EOC in a study involving 261 benign ovarian masses and 229 cases of EOC. However, it is important to note that we presented in our casuistic not only EOC, but a considerable number of non-epithelial, metastatic and borderline ovarian tumors, which reproduces a more realistic scenario of the clinical practice.

One systematic review^22^ showed that the PLR and NLR values in EOC patients deviate from that of healthy controls. However, their diagnostic accuracy remains limited, as their sensitivity and specificity were moderate (55% to 80% of cases of OC detected),^22^ which corroborates our findings. Furthermore, this review^22^ points out that the diagnostic cutoff values are undefined until now.^22^

## Conclusion

The present study has some limitations. Besides its retrospective nature, three different assays were used to measure CA125, and two automated hematology analyzers were used to obtain the preoperative CBC. However, all of these assays were performed at a single institution, and the cutoff applied did not change along the period of the study, with no influence in the medical decision-making. On the other hand, the major strength was our relatively large casuistic, which involved not only EOC, but other less frequent histologic types of ovarian tumors, which may be found in the clinical practice. Finally, we showed that in the preoperative evaluation, no CBC parameter was superior to CA125 in the prediction of the malignancy of ovarian tumors.
